# Neurological complications of SARS-CoV-2 infection among solid organ transplanted patients: does immunosuppression matter?

**DOI:** 10.3389/fneur.2024.1393104

**Published:** 2024-07-04

**Authors:** Federica Avorio, Giovanna Russelli, Giovanna Panarello, Rossella Alduino, Pier Giulio Conaldi, Vincenzina Lo Re

**Affiliations:** ^1^Neurology Service, Department of Diagnostic and Therapeutic Services, IRCCS ISMETT (Istituto Mediterraneo per i Trapianti e Terapie ad Alta Specializzazione), University of Pittsburgh Medical Center Italy (UPMCI), Palermo, Italy; ^2^Department of Research, IRCCS ISMETT, UPMCI, Palermo, Italy; ^3^Department of Anesthesiology and Intensive Care, IRCCS ISMETT, UPMCI, Palermo, Italy

**Keywords:** SARS-CoV-2 infection, neurological manifestations, solid organ transplant, immunosuppression, COVID-19, central nervous system, neurological complications, inflammation

## Abstract

**Introduction:**

SARS-CoV-2 infection can lead to a broad range of neurological manifestations such as olfactory and gustative disorders, myalgias, headache, and fatigue but also more rare and severe neurological pictures such seizures, encephalitis, and cerebrovascular diseases. It is still unknown if the underlying pathophysiological mechanism is the direct cytotoxic effect of the virus on central nervous system or if the related systemic inflammation leads to cerebral suffering and neurological symptoms. Studying neurological manifestations of SARS-CoV-2 infection among solid organ transplant recipients, who take immunosuppressive drugs, may help to shed light on this topic.

**Methods:**

We enrolled a total of 73 solid organ transplantation recipients (kidney, liver, lung, heart and combined) with a history of SARS-CoV-2 infection (in the period between July 2020 and June 2021). We collected all demographic and clinical general information and, through phone interviews, we registered retrospectively the occurrence of neurological symptoms during the acute phase of infection and within the next 6 months.

**Results:**

Approximately 27.4% (20/73) of patients needed hospitalization during the infection, 25.3% (18/73) were treated with oxygen therapy, and only one patient was admitted to the Intensive Care Unit for mechanical ventilation. Almost 74% (54/73) of patients reported at least one neurological symptom/disease. The most frequent neurological complications were myalgia (57.5%), headache (37%), and hyposmia/hypogeusia (37%). Need of oxygen therapy during the SARS-CoV-2 infection was statistically significantly associated to neurological complications (*p*= 0.0344). Pre-infection neurological comorbidities and immunosuppression levels (higher levels of tacrolimus and also being on steroids) did not modify the probability to have neurological manifestations.

**Discussion:**

Frequency of headache was comparable with the same self-reported symptom in the general population, while hyposmia/hypogeusia was more frequent in our cohort of transplant recipients. Higher level of tacrolimus as well as being on steroids did not result protective against neurological manifestation. Lastly neurological symptoms occurred more frequent in more severe cases of infection.

## Introduction

Greater understanding supports the hypothesis that one of the pathophysiological pathways underlying neurological dysfunction, which is frequently observed in severe acute respiratory syndrome coronavirus 2 (SARS-CoV-2) infection, is related to the immunological response triggered by the virus replication instead of a direct cytopathic effect of the virus itself (1). In a few individuals the immune response, necessary in controlling the virus replication, leads to a cytokine storm, a systemic abnormal hyperinflammation that can be detrimental, also locally, to the central nervous system (CNS). Higher levels of circulating pro-inflammatory cytokines, including tumor necrosis factor-alpha (TNFα), interleukin (IL)-1β, IL-6, IL-12, and interferon gamma (INFγ), can cause the disruption of the blood–brain barrier’s integrity, and neurotoxicity (2). The variety of neurological manifestations described in the acute and post-acute phases of SARS-CoV-2 infection in the general population is broad, ranging from self-reported unspecific symptoms (headache, confusion, dizziness, fatigue, anosmia, and ageusia) to more severe and objective signs of central/peripheral nervous system impairment, and syndromes or diseases (seizures, encephalopathy, encephalitis, cerebrovascular diseases) (3, 4). Hypercytokinemia is observed most often in severe coronavirus disease 2019 (COVID-19) patients (5), and patients with severe COVID-19 infection tend to develop more neurological abnormalities compared to those with mild infection (4), suggesting a potential correlation between the hyperinflammatory status and the neurological complications.

It is still unclear whether a therapeutic or chronic pathological status of immunosuppression can worsen or improve clinical outcomes in COVID-19 in the general population. If, on the one hand, being on immunosuppressive drugs can facilitate virus replication as well as the risk of opportunistic infections, on the other, immunosuppression may modulate the exuberant inflammatory response in COVID-19 (6). According to a meta-analysis by Gatti et al. (7), a higher risk of intensive care unit (ICU) admission and occurrence of acute kidney injury was found in solid organ transplant (SOT) recipients; however, no increased risk in mortality was found compared with the general population. Since no study has focused on neurological complications of SARS-CoV-2 infection in transplant recipients, we were interested in understanding how the immunosuppressed status of SOT recipients influences the occurrence of neurological impairment during SARS-CoV-2 infection.

## Materials and methods

We undertook a single center retrospective study of SOT patients with a history of SARS-CoV-2 infection during the second and third waves (July 2020 to June 2021) of the pandemic. The study was approved by our institute’s ethics committee (IRRB/05/21) and was conducted from November 2021 to March 2022. The inclusion criteria were: adults with a status of SOT recipient and a history of SARS-CoV-2 infection occurred at least 6 months before the enrolment time, corresponding with the date when the informed consent was provided. We excluded all patients who did not provide a signed consent form for the study or if clinical information was not available. We enrolled 99 SOT patients, 26 were excluded, and our final cohort was composed of 73 patients. All demographic and clinical information was retrieved by reviewing our electronic medical charts or through phone interviews. When our data were retrieved through the phone call we did not use a structured standardized questionnaire but we just assessed the occurrence of at least one neurological complication reading the whole list of neurological symptoms/signs/syndromes/diseases. When a patient reported a neurological diagnosis we asked for a formal medical report to prove the neurological syndrome or disease. The primary outcome was the occurrence of at least one neurological symptom/sign/syndrome/disease during the acute phase of infection and within the next 6 months; the secondary outcome was to determine whether any demographic (age, sex, body mass index) and/or clinical (type of organ transplant, pre-infection tacrolimus blood level, hospitalization status, non-invasive oxygen therapy need, mechanical ventilation, neurological comorbidities, being on steroids before infection) feature resulted associated with neurological impairment.

All included cases had a confirmed SARS-CoV-2 infection according to a positive molecular/antigenic virological test with a registered date of first positivity (8). COVID wave dates were defined according to the Italian National Institute of Health (ISS), as reported by Beretta et al. (9). Neurological symptoms were self-reported and we included: headache, hyposmia/hypogeusia, myalgia, affective and behavioral impairment, visual impairment, impaired sensation, dizziness; neurological signs/syndromes/diseases, including ataxia, seizures, status epilepticus, meningoencephalitis, delirium, coma, ischemic or hemorrhagic stroke, weakness, neuropathic pain, were considered if reported on the medical chart and diagnosed by a physician. We explored all neurological symptoms and signs mainly considered in the Neuro-COVID Italy study (9). At the time of positivity, the chronic immunosuppression regimen included prednisone, mycophenolate mofetil, and tacrolimus for kidney, heart and combined (kidney-pancreas) transplants; liver transplant patients received only tacrolimus, while lung transplant patients received tacrolimus plus steroids. Only two patients (one liver and one kidney transplanted) were immunosuppressed with everolimus. The percentage of our sample that received the first dose of messenger ribonucleic acid (mRNA) vaccine at the time of infection was irrelevant since we started the anti-SARS-CoV-2 vaccination campaign for this high-risk population in April 2021. Also, it has been noted that in this population Pfizer-BioNTech BNT162b2 mRNA vaccine induces an impaired anti-SARS-CoV-2 humoral and cellular immune response (10).

Continuous and categorical variables are expressed as mean with standard deviation and as frequency with percentage, respectively. To compare continuous variables, Wilcoxon tests or t-test were used when appropriate. Chi-square tests were used to compare categorical variables among groups. To explore the differences between the two groups (patients with and patients without neurological manifestations), logistic regression models were used. Levels of significance were set at a *p* < 0.05. Statistical analyses were done with SAS software version 9.4.

## Results

Table 1 lists all demographic and clinical features of the entire cohort of enrolled patients (first column); we also report the same variables for two patient groups categorized according to our first outcome (occurrence of neurological manifestations during coronavirus infection; second and third columns). The SOT cohort of patients included mainly kidney (45.2%) and liver (42.5%) transplants, but also lung (6.8%), heart (1.4%), and combined (4.1%) transplant patients. The median time from transplant to infection in years was 5.5 (interquartile range: 3–11) for the whole cohort, with no differences between the two categories of patients (*p* = 0.4667). Approximately 27.4% (20/73) of our patients were hospitalized during the infection, 25.3% (18/73) needed oxygen (O_2_)-therapy, and only one patient was admitted to the ICU for mechanical ventilation (Table 2). Almost 74% (54/73) of patients from our transplant cohort complained of at least one neurological symptom/sign during SARS-CoV-2 infection and within the next 6 months. The most frequent neurological complications (Table 3) during coronavirus infection were self-reported and aspecific: myalgia, headache, and hyposmia/hypogeusia, respectively, 57.5%, 37%, and 37%. Looking at potential predisposing demographic features or comorbidities associated with neurological impairment during SARS-CoV-2 infection, we found no statistically significant association except for need of O_2_-therapy (*p =* 0.0344) and a weak correlation with being hospitalized (*p =* 0.0552). Pre-infection neurological comorbidities and immunosuppression levels (higher levels of tacrolimus) did not increase or protect against neurological manifestations. An immunosuppressive regimen including steroids, at the time of SARS-CoV-2 infection, shows a positive trend association (*p =* 0.0768) with neurological complications occurrence.

**Table 1:** Demographic and clinical data of SOT patients with a history of SARS-CoV-2 infection.

Baseline features	All patients (*n* = 73)	Patients with neurological manifestations (*n* = 54)	Patients with no neurological manifestations (*n* = 19)	*P*_value
Age in years, mean (SD)	53.43 (15.87)	53.15 (14.89)	54.21 (18.81)	0.5845
Male sex, *n* (%)	46 (63%)	34 (62.9%)	12 (63.1%)	0.9879
**Organ transplant, *n* (%)**				
KTx	33 (45.2%)	26 (48.1%)	7 (36.8%)	0.5684
OLTx	31 (42.5%)	21 (38.9%)	10 (52.6%)	
LTx	5 (6.8%)	3 (5.6%)	2 (10.5%)	
HTx	1 (1.4%)	1 (1.8%)	0	
Combined transplant	3 (4.1%)	3 (5.6%)	0	
**Time from transplant to infection in years, median (IQR)**				
BMI mean (SD)	27.56^*^ (4.92)	28.03^**^(5.38)	26.24^***^(3.04)	0.2758
Last tacrolimus blood level before infection (ng/mL). mean (SD)	5.78^****^(2.91)	6.02^***^ (3.01)	4.66^*^ (2.09)	0.2016
Neurological comorbidities	24 (33.3%)	17 (31.5%)	7 (38.9%)^*****^	0.5637
Immunosuppression regimen including steroids	37 (51.4%)	31 (57.4%)	6 (33.3%)^*****^	0.0768

Demographic and clinical data of all SOT patients with a history of SARS-CoV-2 infection, and from categorized patients as having (*n* = 54) or not (*n* = 19) at least one neurological symptom (primary endpoint) are presented in order to detect specific associated factors. ^*^8 missing values. ^**^6 missing values. ^***^2 missing values. ^****^10 missing values. ^*****^1 missing values.

**Table 2:** Clinical outcomes related to the SARS-CoV-2 infection in our whole cohort of SOT patients and from categorized patients as having (*n* = 54) or not (*n* = 19) at least one neurological symptom (primary endpoint) are presented in order to detect associated factors.

Clinical outcomes at infection time	All patients (*n* = 73)	Patients with neurological manifestations (*n* = 54)	Patients with no neurological manifestations (*n* = 19)	*P*_value
**Outcomes *n* (%)**				
Hospitalized	20 (27.4%)	18 (33.3%)	2 (10.5%)	0.0552
Oxygen (O_2_-therapy)	18 (25.3%)	17 (31.5%)	1 (5.9%)^***^	**0.0344**
No-hospitalization/No-O_2_-therapy	49 (69.0%)	33 (61.1%)	16^***^ (94.1%)	0.1146
No-hospitalization/non-invasive O_2_-therapy	3 (4.2%)	3 (5.6%)	0	
Hospitalization/without O_2_-therapy	5 (7.0%)	4 (7.4%)	1^***^ (5.9%)	
Hospitalization with non-invasive O_2_-therapy	13 (18.3%)	13 (24.1%)	0	
ICU admission/mechanical ventilation	1 (1.4%)	1 (1.8%)	0	

Value in bold indicate statistically significant differences; ^***^ 2 missing values.

**Table 3:** Clinical features of SOT patients with SARS-CoV-2 infection and at least one neurological symptom cohort (*n* = 54).

Neurological symptoms/signs/syndromes	% Neurological cohort (54)	% of Whole cohort (73)
Headache	27 (50%)	37.0%
Hyposmia/Hypogeusia	27 (50%)	37.0%
Myalgia	42 (77.8%)	57.5%
Cognitive impairment	17 (31.5%)	23.3%
Affective and behavioral impairment	10 (18.5%)	13.7%
Visual impairment	2 (3.7%)	2.7%
Ataxia	0	
Seizures	0	
Status epilepticus	0	
Meningoencephalitis	0	
Delirium	0	
Coma	1 (1.8%)	1.4%
Ischemic stroke	0	
Intracranial bleeding	0	
Weakness	1 (1.8%)	1.4%
Impaired sensation	2 (3.7%)	2.7%
Neuropathic pain	0	
Dizziness	5 (9.3%)	6.8%

Neurological symptoms were self-reported; neurological signs and syndromes were considered when reported on medical charts and evaluated by physicians.

## Discussion

How chronic immunosuppression influences the nervous system impairment seen during SARS-CoV-2 infection in immunosuppressed patients has yet to be addressed. Understanding the role of immunosuppression might provide clues to the pathophysiological mechanisms responsible for the known neurological damage associated with the acute phase of coronavirus infection, inflammatory processes being the most relevant (11), but also address the best immunosuppression regimen management during the infection. In our study, we found no substantial differences in the frequency and types of neurological impairment in our population of immunosuppressed patients compared to a general population as described in international registries (12, 13). More precisely, myalgia (57.5%) was the most common neurological complaint in our cohort, with an overlapping frequency reported by Beghi et al. for non-hospitalized patients from a general population (51.9%). Also, headache was the second most common self-reported symptom in our SOT patients with recent SARS-CoV-2 infection (37.0%), as well as in the multicenter cohort study on COVID-19 hospitalized patients described by Chou et al. (37%), while hyposmia/hypogeusia was more frequent in our immunosuppressed patients (37.0% vs. 26%) than in the general population considered in the cited study.

We did not find that pre-infection higher blood levels of the calcineurin-inhibitor tacrolimus, the most common immunosuppressive drug in our population, or being on steroid therapy was protective for neurological outcomes. This finding is consonant with the largest cohort studies on chronic immunosuppressed patients that describe the same general clinical outcomes (mechanical ventilation, in-hospital death, length of stay) after COVID-19 in the general population (6, 7), and may indirectly suggest that the pathophysiology of central nervous system impairment in COVID-19 implies multiple pathways, and not only hyper-inflammation (12). Last, neurological symptoms and signs are more likely to occur in more severe cases of infection, confirming an already known correlation. This can also explain the weak positive association between being on steroids at the time of SARS-CoV-2 infection and neurological complications occurrence: though steroids are strongly recommended for COVID-19 associated acute respiratory distress syndrome (14), there is evidence they could be harmful in earlier stages of infection delaying the viral clearance and increasing the viral load (15).

Our study has limitations, with the absence of a control group, the small sample size, and the retrospective-monocentric study design so we cannot exclude a recall bias mainly for self-reported neurological symptoms. However, the principal message is original in the literature, confirming that neurological impairment during SARS-CoV-2 infection in SOT occurs with the same features as in the general population.

## Data availability statement

The original contributions presented in the study are included in the article/supplementary material, further inquiries can be directed to the corresponding author.

## Ethics statement

The studies involving humans were approved by Comitato Etico Locale ISMETT, IRRB 05/2021. The studies were conducted in accordance with the local legislation and institutional requirements. The participants provided their written informed consent to participate in this study.

## Author contributions

FA: Writing – original draft, Writing – review & editing, Conceptualization, Data curation, Investigation, Validation. GR: Data curation, Methodology, Writing – original draft. GP: Data curation, Investigation, Methodology, Resources, Writing – original draft. RA: Data curation, Formal analysis, Methodology, Writing – original draft. PC: Supervision, Validation, Writing – review & editing. VL: Supervision, Validation, Writing – review & editing, Investigation, Project administration.
